# Treatment of cutaneous vasculitis

**DOI:** 10.3389/fmed.2022.1059612

**Published:** 2022-11-18

**Authors:** Robert G. Micheletti

**Affiliations:** ^1^Department of Dermatology, Perelman School of Medicine, University of Pennsylvania, Philadelphia, PA, United States; ^2^Department of Medicine, Perelman School of Medicine, University of Pennsylvania, Philadelphia, PA, United States

**Keywords:** vasculitis, cutaneous vasculitis, treatment, management, skin

## Abstract

Cutaneous vasculitis encompasses a spectrum of disease states, with varied morphology, severity, and potential for systemic involvement. Even vasculitis which is skin-limited can have a significant quality-of-life impact, necessitating treatment. This manuscript summarizes the available evidence for management of various types of skin-limited vasculitis and provides a proposed therapeutic ladder based on published studies and expert opinion.

## Principles of therapy

Vasculitis of the skin (cutaneous vasculitis) encompasses a spectrum of disease states, ranging from cutaneous manifestations of systemic vasculitis (e.g., purpura in a patient with granulomatosis with polyangiitis), to skin-limited variants of systemic vasculitis (e.g., cutaneous polyarteritis nodosa), to various types of single-organ vasculitis found only in the skin (e.g., erythema elevatum diutinum) (1, 2). Whether systemic or skin-limited, the severity of symptoms also varies. That severity—and the presence or absence of internal organ involvement—guides therapy.

In cases of systemic disease where the skin is involved, appropriate systemic treatment will often, though not always, effectively manage the skin. In cases where the skin is primarily affected, specific therapies with activity in the skin and a favorable risk-benefit profile are preferred. Finally, in many cases of skin-limited vasculitis, no treatment at all may be necessary, provided the process is minimally symptomatic and self-limited.

Ultimately, the types of vasculitis affecting the skin are varied, and the data guiding their management are poor, based primarily on case series and expert opinion (Table 1). Yet, the importance of effective management of cutaneous vasculitis is clear: A survey of patients with cutaneous manifestations of vasculitis (all major types) demonstrated that cutaneous vasculitis is associated with diminished health-related quality-of-life across multiple domains, showing a significant impact on patient symptoms, wellbeing, and self-perception of health (3). For this reason, effective management of cutaneous vasculitis, in all its forms, is vital.

**Table 1:** Proposed therapeutic ladder for patients with various types of skin-limited vasculitis.

**Disorder**	**First-line treatment**	**Second-line treatment**	**Third-line treatment**
**Cutaneous IgM/IgG Immune Complex Vasculitis** (primary cutaneous small vessel vasculitis/cutaneous leukocytoclastic angiitis/hypersensitivity vasculitis)	Eliminate/treat potential triggers Supportive care (rest, elevation) NSAIDs Topical steroids	Colchicine (0.6 mg BID) (13–15) Dapsone (50–200 mg/day) (16) AZA (2 mg/kg/day) (14, 17) CS (e.g., prednisone up to 40-60 mg/day tapered over 3-4 weeks) (6, 7)	COL + DAP (12)
			MTX (18)
			MYC (19)
			CSA (20)
			CYC
			Rituximab (21)
			Infliximab (22)
			IVIg (23)
**Skin-limited IgA vasculitis** (Henoch–Schönlein purpura)	Supportive care NSAIDs Topical steroids	Colchicine Dapsone (24) AZA (25) CS	Rituximab (55)
			MYC (56)
			CSA (57)
			CYC (57)
			PEX (58)
**Cutaneous polyarteritis nodosa**	Supportive care (31) NSAIDs (31) Colchicine (31) Dapsone (31) CS (0.5–1 mg/kg/day tapered over months) (28)	AZA (2 mg/kg/day) (31) MTX (15–25 mg/wk) (35)	TNF-αinhibitors (36, 37)
			MYC
			CYC (38)
			HCQ (32)
			IVIg (39)
**Urticarial vasculitis**	Antihistamines (42) NSAIDs (42) Dapsone (100–200 mg/day) (42) CS (42)	Colchicine (0.6 mg BID–TID) (42) HCQ (42) MYC (42) AZA (42) MTX (42) Dapsone + pentoxifylline (42)	Omalizumab (42)
			Anakinra/Canakinumab (42)
			Rituximab (42)
			CYC (42)
			CSA (42)
			IVIg (42)
			Tocilizumab (42)
**Cryoglobulinemic vasculitis** (skin-predominate)	Direct-acting antivirals (e.g., sofosbuvir-velpatasvir) if HCV+ (59) Supportive care	Colchicine (43, 44) Dapsone	Rituximab (60)
			CS (58)
			IVIg (61)
			PEX (62)
**Erythema elevatum diutinum**	Dapsone (45) Intralesional CS (45) NSAIDs (45)	Colchicine (45) Chloroquine (45) Tetracyclines± niacinamide (45)	MYC (45)
			MTX (45)
			CS (45)
**Acute hemorrhagic edema of infancy**	Supportive care (63) Antihistamines	CS (46)	
**Nodular vasculitis** (erythema induratum)	Eliminate/treat potential triggers (anti-TB therapy, if indicated) Supportive care NSAIDs Potassium iodide (300 mg TID) (47, 48)	Colchicine (49)	CS (49)
			MYC (49)

AZA, azathioprine; BID, twice daily; CS, corticosteroids; COL, colchicine; CSA, cyclosporine; CYC, cyclophosphamide; DAP, dapsone; HCQ, hydroxychloroquine; HCV, hepatitis C; Ig, immunoglobulin; IVIg, intravenous immunoglobulin; MTX, methotrexate; MYC, mycophenolate mofetil; NSAIDs, nonsteroidal anti-inflammatory drugs; PEX, plasma exchange; TID, three times a day.

## Cutaneous IgM/IgG immune complex vasculitis

Cutaneous IgM/IgG immune complex vasculitis, as defined in the dermatologic addendum to the Chapel Hill Consensus Conference nomenclature, is a type of immune complex-mediated small vessel vasculitis which is limited to the skin (2). This entity is the most common subgroup of patients presenting with palpable purpura on the lower extremities, referred to elsewhere (and less precisely) in the literature as “cutaneous leukocytoclastic angiitis,” “cutaneous small vessel vasculitis,” “hypersensitivity vasculitis,” or “leukocytoclastic vasculitis.”

At the time of initial presentation, it generally is not yet clear whether small vessel vasculitis of the skin is associated with systemic vasculitis, secondary to some underlying trigger, or possibly consistent with cutaneous IgM/IgG immune complex vasculitis instead. A systematic approach to evaluation is needed to differentiate these possibilities (4). Biopsies for routine processing and direct immunofluorescence studies help confirm the diagnosis and rule out other conditions, like IgA vasculitis (which carries an increased risk of systemic involvement), while thorough examination, review of systems, and stepwise laboratory testing help identify those patients with internal organ involvement or important underlying disease states.

The need for treatment depends on the presence of underlying disease states, if any, as well as the chronicity and severity of the vasculitis. Because most initial episodes of small vessel vasculitis presenting in the skin are skin-limited and self-limited, resolving within 3–4 weeks (5), systemic therapy is not needed acutely in most cases. Identifiable triggers should be treated or removed. Simple measures, like rest, elevation, or compression, and topical steroids for itch relief, may be all that is required. More than half of patients require no systemic treatment at all (6).

However, those with severe, chronic (lasting longer than 4 weeks), or recurrent disease should receive treatment, even if the lesions are relatively asymptomatic, because of the quality-of-life impact (3). Unfortunately, no robust literature is available to guide management; treatment recommendations are based on case reports, case series, and expert opinion (Table 2). For those with painful, ulcerative, or otherwise highly symptomatic disease, oral glucocorticoids (e.g., 0.5–1 mg/kg/day prednisone equivalent) may be appropriate. In most cases, systemic glucocorticoids help speed resolution and can be tapered successfully over 3–6 weeks (6, 7). However, not all patients respond adequately to systemic glucocorticoids, or they experience flares with attempted taper. Given the many associated side effects, systemic glucocorticoids are not an appropriate long-term option for treatment of cutaneous vasculitis (8–11). Therefore, those with chronic/recurring vasculitis, and those with vasculitis which flares with attempted taper, should initiate an appropriate steroid-sparing agent.

**Table 2:** Expanded treatment ladder for skin-limited cutaneous small vessel vasculitis.

**Clinical scenario**	**Treatment**
Initial episode^*^ ~90% of patients	Eliminate underlying cause, e.g., drug, infection (if identified) Rest, elevation, compression NSAIDs for pain; topical steroids for itch If severe, consider systemic steroids (e.g., prednisone up to 40-60 mg daily tapered over 3-4 weeks) (6, 7)
Chronic/recurrent^†^ ~10% of patients	First-line: Colchicine 0.6 mg twice daily (13–15) Dapsone 100-150 mg daily (16) Azathioprine 2 mg/kg daily (14, 17) Systemic steroids (used sparingly during flares) (6, 7)
Severe or refractory	Second-line: Combination therapies, e.g., dapsone plus colchicine (12) Mycophenolate mofetil 2-3 g daily (19) Methotrexate 15-25 mg weekly (18) Hydroxychloroquine 200-400 mg daily‡ (42) Pentoxifylline 400 mg three times daily‡ (42) Severe disease only: Rituximab 1 g intravenous on days 1 and 15 (21) Infliximab 5-10 mg/kg every 4-8 weeks (maintenance) (22) Cyclosporine 2.5-5 mg/kg daily in divided doses (20) Intravenous immunoglobulin 2 g/kg monthly, divided over 2-4 days (23)

* Most episodes resolve within 3-4 weeks;

† Persisting or recurring over > 4 weeks;

^‡^Usually in combination with other agents.

Lacking high-quality data, there is considerable practice variation regarding the choice of steroid-sparing agents. Based on available studies and expert opinion, colchicine, dapsone, and azathioprine are reasonable initial options (12). These drugs are relatively safe and well-tolerated and are commonly used for cutaneous vasculitis. However, often more than one drug must be tried in order to find that which is most effective and best tolerated.

Colchicine (0.6 mg twice daily) has been reported effective for skin and joint symptoms in open-label series (13, 14). However, it showed no benefit compared to placebo in a small, 1 month long randomized controlled trial (15). Gastrointestinal side effects (abdominal discomfort, loose stools) are limiting in some patients.

Dapsone (typically 100–150 mg/day) is another option, supported by case reports and expert opinion (16). Testing for glucose-6-phosphate dehydrogenase deficiency must be performed prior to initiation, and routine monitoring should be conducted to monitor for anemia. Methemoglobinemia is a rare side effect of dapsone which should be considered in patients reporting low pulse oximetry readings or dyspnea. Dapsone is sometimes combined with colchicine for added benefit in cutaneous vasculitis (12).

Azathioprine (usually 2 mg/kg/day divided twice daily) has been reported to be efficacious for cutaneous vasculitis and is frequently used for treatment of various systemic vasculitides (14, 17). Screening for reduced thiopurine S-methyltransferase (TPMT) activity is common practice for identifying and excluding patients with increased risk of leukopenia. Hepatic injury, hypersensitivity, and infectious complications are other risks.

Alternative options can be considered in those who fail to respond to the above therapies. These include methotrexate (15–25 mg/week) (18) and mycophenolate mofetil (2–3 g/day) (19) and, in rare instances, more aggressive therapies, such as cyclosporine (20), cyclophosphamide, rituximab (21), infliximab (22), or intravenous immune globulin (23). Absent high-quality data supporting these agents, disease severity and potential drug toxicities should be carefully weighed in determining appropriate next steps.

## Skin-limited IgA vasculitis

IgA vasculitis (otherwise know as Henoch-Schonlein purpura) is an IgA-mediated systemic vasculitis. While the presentation of IgA vasculitis in the skin may be indistinguishable from that of cutaneous IgM/IgG immune complex vasculitis, patients with IgA vasculitis are much more likely to have gastrointestinal, joint, or renal manifestations. Fortunately, the overall prognosis is favorable, and the condition is frequently skin-limited.

As with other forms of vasculitis, the treatment of IgA vasculitis is dictated by the extent and severity of the condition. Those with IgA vasculitis which is skin-limited should be treated similarly to patients with cutaneous IgM/IgG immune complex vasculitis. Because IgA vasculitis is often self-limited, resolving over weeks to months, nothing more than supportive care may be needed for management of a minimally symptomatic initial episode. For symptomatic or chronic/recurring lesions, colchicine and dapsone are reasonable options, as is azathioprine (24, 25). Because of the association of IgA vasculitis with glomerulonephritis, systemic glucocorticoids have been evaluated for prevention of renal complications but have not been shown to be beneficial as prophylaxis (26).

## Cutaneous polyarteritis nodosa

Cutaneous polyarteritis nodosa (cPAN) is a skin-predominate medium-sized vessel vasculitis representing perhaps 4% of polyarteritis nodosa cases (27). Patients typically present with fixed livedo racemosa (so-called “starburst” livedo) and tender subcutaneous nodules involving the lower legs. Less commonly, the arms and trunk are involved. Ulcerations are seen in up to half of patients (28). Mild systemic symptoms are common, including fever, myalgias, arthralgias, and peripheral neuropathy in the territory of current or prior skin lesions (29). However, the presence of more prominent constitutional, visceral, or neurological symptoms suggests a diagnosis of systemic PAN instead. A chronic, relapsing course is typical of cPAN, though available data suggest the risk of evolution to systemic PAN is extremely low (30).

Limited data are available to guide the treatment of cPAN, but like other forms of skin-limited vasculitis, disease severity should guide management. NSAIDs, rest, and elevation may improve mild symptoms (31), but a trial of colchicine (0.6mg twice daily) or dapsone (50–150mg daily) is appropriate for most patients presenting with subcutaneous nodules (31). These agents are commonly used for treatment of skin-limited vasculitis and have a favorable risk profile. Other non-immunosuppressive agents (e.g., sulfapyridine, hydroxychloroquine, pentoxifylline) are supported by case reports and series and are reasonable options unless symptoms are severe (32–34).

Systemic corticosteroids may be indicated during acute flares, especially for management of pain, ulceration, or systemic symptoms, such as arthralgias, paresthesias, and malaise. Prednisone 30mg per day or equivalent is generally sufficient, but higher doses (e.g., 1 mg/kg prednisone daily) should be considered in the setting of more severe symptoms (e.g., digital ischemia), or if lower doses produce an insufficient response (28). Once remission has been achieved, systemic steroids should be tapered slowly. Coadministration of a steroid-sparing agent may facilitate successful taper.

For those with severe or treatment-refractory cPAN, various immunosuppressive agents should be considered. Azathioprine (2mg/kg daily) (31) or methotrexate (15–25mg weekly) (35) are commonly used. Patients with severe, painful, ulcerative disease who fail to respond to these agents may respond to therapy with a TNFa inhibitor (36, 37), cyclophosphamide (38), or intravenous immunoglobulin (39). In cPAN, ulcerative disease correlates with a relapsing course and the need for more aggressive therapy (40).

## Alternative regimens and special situations

Therapeutic options for less common types of skin-limited vasculitis are even less well defined, drawing mostly upon the experience with other types of cutaneous or systemic vasculitis. Nevertheless, based on limited data, certain therapies may be more effective in specific disorders, as noted below and in Table 1.

## Urticarial vasculitis

Urticarial Vasculitis is a condition consisting of hive-like skin lesions with histologic features of leukocytoclastic vasculitis. Urticarial vasculitis with normal complement levels is best thought of as a variant of skin-limited cutaneous small vessel vasculitis where the lesions just happen to look hive-like. By contrast, those with low complement levels (hypocomplementemic urticarial vasculitis) are much more likely to have systemic manifestations of disease (such as musculoskeletal, gastrointestinal, or pulmonary symptoms) and to meet criteria for systemic lupus (41).

No trials have been performed to evaluate treatment options for urticarial vasculitis. Because diagnosis and classification of this condition can be difficult, existing case series may include a range of patients, including some with chronic idiopathic urticaria and related disorders.

Antihistamines may reduce swelling and pain associated with skin lesions. Oral steroids are often beneficial but are not appropriate long-term therapy. Dapsone with or without pentoxifylline, hydroxychloroquine, colchicine, and mycophenolate mofetil have been reported to be efficacious in some cases. Omalizumab, anakinra, canakinumab, and rituximab are options for management of recalcitrant hypocomplementemic urticarial vasculitis. A systemic review of treatments identifies a range of potential therapies with variable supporting evidence (42) (Table 1).

## Skin-predominant cryoglobulinemic vasculitis

Cryoglobulinemic vasculitis, resulting from the presence of type II or III cryoglobulins, typically affects the skin, joints, peripheral nervous system, and kidneys. Clinical findings reflect a mixed small and medium-sized vessel vasculitis. Characteristic cutaneous manifestations include palpable purpura on the lower extremities, livedo reticularis, retiform purpura, and necrosis/ulceration.

Some patients with cryoglobulinemic vasculitis have only mild manifestations; in fact, a skin-limited subtype is recognized in the dermatologic addendum to the Chapel Hill Consensus Conference Nomenclature (2). Patients with cutaneous manifestations (e.g., palpable purpura) but lacking significant systemic findings may respond well to agents like colchicine or dapsone (43, 44). Such patients should be monitored over time for disease response and progression. Those with underlying hepatitis C should receive appropriate antiviral therapy.

## Erythema elevatum diutinum

Erythema elevatum diutinum (EED) is a rare, chronic type of cutaneous vasculitis characterized by red-brown papules and plaques which favor acral and extensor surfaces, with histologic features showing leukocytoclastic features and fibrosis. Though it is usually skin-limited, underlying joints and the eyes may be involved.

Dapsone is the therapy of choice for EED. Other options include intralesional steroids, NSAIDs, tetracyclines +/– niacinamide, chloroquine, and colchicine. Treatment of any underlying autoimmune, inflammatory, or hematologic disorder is also advised (45).

## Acute hemorrhagic edema of infancy

Acute hemorrhagic edema of infancy (AHEI) is a rare form of small vessel vasculitis affecting children under 24 months of age and characterized by annular or targetoid purpuric plaques favoring the face, ears, and extremities. While the course is benign, with spontaneous resolution typically occurring within 1–3 weeks, fever, arthralgias, abdominal pain, and glomerulonephritis may occur.

Treatment of AHEI is supportive. Antihistamines and systemic steroids may improve acute symptoms (46). Rarely are therapies indicated on a chronic basis.

## Nodular vasculitis (erythema induratum)

Nodular vasculitis is a lobular panniculitis with vasculitis of small and/or medium-sized vessels in the panniculus. When occurring in the setting of tuberculosis, it may be referred to as erythema induratum (of Bazin). The process is skin-limited/skin-predominate but may be associated with peripheral neuropathy and symptoms related to an underlying/associated disorder, if any.

Initial therapy of nodular vasculitis/erythema induratum should include withdrawal or treatment of any underlying trigger. If associated with tuberculosis, it should be treated with standard anti-tuberculous therapy. Additional options for therapy include nonsteroidal anti-inflammatory drugs and supportive care. Case series describe rapid improvement with potassium iodide (300mg three times daily) (47, 48). Other options (e.g., colchicine, systemic steroids, mycophenolate mofetil) may also be beneficial (49).

## Conclusion and future directions in vasculitis therapy

Presently, the data guiding management of cutaneous vasculitis are extremely limited. Because many vasculitis subtypes are quite rare, multi-institutional collaborations will be necessary to pool combined experiences and conduct high-quality studies.

A multicenter, randomized trial is currently underway comparing colchicine, dapsone, and azathioprine for management of patients with one of the more common subtypes of isolated cutaneous vasculitis (primary cutaneous small vessel vasculitis, skin-limited IgA vasculitis, and cutaneous polyarteritis nodosa; ClinicalTrials.gov Identifier: NCT02939573) (50).

Vasculitis type, severity, and patient comorbidities determine treatment selection, with the goal of inducing and maintaining disease remission while minimizing drug toxicity. Recent trends in the management of systemic vasculitis demonstrate a shift away from broadly immunosuppressive regimens toward more targeted therapies, based on an improved understanding of disease pathogenesis. Terminal complement inhibition for management of ANCA-associated vasculitis with avacopan (51) and blockade of interleukin-5 for treatment of eosinophilic granulomatosis with polyangiitis with mepolizumab illustrate this trend (52).

The Cutaneous Transcriptomics in Systemic Vasculitis (CUTIS) study is another multicenter collaborative effort to evaluate the histopathologic and transcriptomic features of systemic, and skin-limited, vasculitides through analysis of blood and lesional skin biopsies (NCT03004326). Observations of cutaneous vasculitis in the setting of COVID-19 (53) and in the recently described VEXAS (vacuoles, E1 enzyme, X-linked, autoinflammatory, somatic) syndrome (54), illustrate the evolving nature of the field. Information gained from these and other studies which enhances understanding of disease pathophysiology may lead to new, targeted approaches to disease management.

## Author contributions

RM drafted the manuscript and takes responsibility for its content.

## Conflict of interest

The author declares that the research was conducted in the absence of any commercial or financial relationships that could be construed as a potential conflict of interest.

## Publisher's note

All claims expressed in this article are solely those of the authors and do not necessarily represent those of their affiliated organizations, or those of the publisher, the editors and the reviewers. Any product that may be evaluated in this article, or claim that may be made by its manufacturer, is not guaranteed or endorsed by the publisher.
